# PTSD and Depressive Symptoms as Potential Mediators of the Association between World Trade Center Exposure and Subjective Cognitive Concerns in Rescue/Recovery Workers

**DOI:** 10.3390/ijerph17165683

**Published:** 2020-08-06

**Authors:** Ankura Singh, Rachel Zeig-Owens, Laura Rabin, Theresa Schwartz, Mayris P. Webber, David Appel, David J. Prezant, Charles B. Hall

**Affiliations:** 1 The Bureau of Health Services and the FDNY World Trade Center Health Program, Fire Department of the City of New York, Brooklyn, New York, NY 11201, USA; ankura.singh@fdny.nyc.gov (A.S.); rachel.zeig-owens@fdny.nyc.gov (R.Z.-O.); theresa.schwartz@fdny.nyc.gov (T.S.); Mayris.Webber@fdny.nyc.gov (M.P.W.); david.appell@fdny.nyc.gov (D.A.); david.prezant@fdny.nyc.gov (D.J.P.); 2 Pulmonary Medicine Division, Department of Medicine, Montefiore Medical Center and Albert Einstein College of Medicine, Bronx, New York, NY 10467, USA; 3 Division of Epidemiology, Department of Epidemiology and Population Health, Albert Einstein College of Medicine, Bronx, New York, NY 10461, USA; 4 Department of Psychology, Brooklyn College and The Graduate Center of CUNY, Brooklyn, New York, NY 11210, USA; lrabin@brooklyn.cuny.edu; 5 Division of Biostatistics, Department of Epidemiology and Population Health, Albert Einstein College of Medicine, Bronx, New York, NY 10461, USA; 6 Saul R. Korey Department of Neurology, Albert Einstein College of Medicine, Block 312, 1300 Morris Park Avenue, Bronx, New York, NY 10461, USA

**Keywords:** occupational exposure, mental health, stress disorders, post-traumatic, epidemiological studies, cognitive dysfunction

## Abstract

We observed that World Trade Center (WTC) exposure, post-traumatic stress disorder (PTSD) symptoms and depressive symptoms were associated with subjective cognitive concerns in Fire Department of the City of New York (FDNY) rescue/recovery workers. This follow-up study examined whether PTSD symptoms and/or depressive symptoms mediate the observed association between WTC exposure and subjective cognitive concerns. We included WTC-exposed FDNY workers who completed the Cognitive Function Instrument (CFI), measuring self-perceived cognitive decline (*N* = 9516). PTSD symptoms and depressive symptoms were assessed using the PCL-S and CES-D, respectively. Multivariable linear regression estimated the association between WTC exposure and CFI score, adjusting for confounders. Mediation analyses followed the methods of Vanderweele (2014). Participants’ average age at CFI assessment was 56.6 ± 7.6 years. Higher-intensity WTC exposure was associated with worse CFI score, an effect that was entirely mediated by PTSD symptoms (%mediated: 110.9%; 95%CI: 83.1–138.9). When substituting depressive symptoms for PTSD symptoms, the WTC exposure–CFI association was largely mediated (%mediated: 82.1%; 95%CI: 60.6–103.7). Our findings that PTSD symptoms and depressive symptoms mediate the association between WTC exposure and subjective cognitive concerns indicate that in the absence of these symptoms, WTC exposure in rescue/recovery workers would not be associated with subjective cognition. Interventions targeting PTSD and depression may have additional value in mitigating cognitive decline in WTC-exposed populations.

## 1. Introduction

Self-perceived decline in cognitive functioning is a common experience for older adults and has, in older populations, been linked to future pathologic cognitive decline and dementia [1,2,3,4]. Recent data from the Centers for Disease Control and Prevention (CDC) show that the prevalence of self-reported worsening confusion or memory loss in the past 12 months in US adults aged 45 years and older is between 11–12% [5]. Previously, we reported that exposure to the World Trade Center (WTC) disaster site, post-traumatic stress disorder (PTSD) symptoms, and depressive symptoms were associated with subjective cognitive concerns in Fire Department of the City of New York (FDNY) rescue/recovery workers [6]. Our findings were consistent with those from a study of non-FDNY WTC responders, which reported that WTC exposure and PTSD symptoms were associated with worse performance on an objective cognitive measure [7]. They also accord with a large body of research showing PTSD symptoms and depressive symptoms to be risk factors for cognitive dysfunction, both in studies of veterans [8,9,10] and in general populations [11,12,13]. PTSD has been linked to reduced cognitive function even in studies of non-veteran trauma survivors in which trauma-exposed comparison groups without PTSD were used, such as natural disaster survivors or war victims [14,15]. Individuals with PTSD showed deficits in several neurocognitive domains including attention, learning, and processing speed [14,15]. While the above analyses involved objective cognitive assessments, the literature contains evidence that PTSD symptoms and depressive symptoms are associated with greater subjective cognitive concerns as well [16,17,18].

Previous analyses conducted in the FDNY WTC-exposed cohort found that individuals with higher levels of WTC exposure had an increased risk of certain mental health conditions, including PTSD and depression [19,20,21,22]. Longitudinal data through September 2010 illustrated that firefighters who arrived at the WTC site on the morning of 9/11 consistently had higher levels of probable PTSD compared to those who arrived to work at the site at later times [22]. In another investigation, longitudinal data through September 2015 showed persistently elevated levels of probable PTSD and probable depression in rescue/recovery workers who had an early WTC site arrival time [20].

In this study, we further explore the relationship between WTC exposure and subjective cognitive function by assessing whether PTSD symptoms and depressive symptoms act as mediators of the association between WTC exposure intensity and subjective cognitive concerns. Our hypothesis is that the mental health symptoms largely mediate this association.

## 2. Materials and Methods

### 2.1. Study Population

Firefighters and emergency medical service providers (EMS) who were actively employed by FDNY on 9/11/2001 (9/11) and who initially arrived to work at the WTC site between 9/11 and 9/24/2001 made up the source population (*N* = 11,483). Shortly after 9/11, the FDNY WTC Health Program instituted regular and ongoing health monitoring examinations, which include self-administered physical and mental health questionnaires, for WTC-exposed workers. The Cognitive Function Instrument (CFI), a self-administered measure of subjective cognitive and functional change [23,24,25], was added to the FDNY WTC monitoring questionnaire in March of 2018. Those who underwent a routine health monitoring examination, and therefore completed the CFI, between 3/1/2018 and 2/29/2020, formed the final study population (*N* = 9516). Participants provided written informed consent, and the Montefiore Medical Center/Albert Einstein College of Medicine Institutional Review Board approved this study (02-02-041E).

### 2.2. Participant Characteristics

We obtained participants’ demographic characteristics and work assignments on 9/11 (firefighter or EMS) from the FDNY employee database, and their highest education level and smoking status from their health questionnaires. If participants completed more than one questionnaire during the 3/1/2018-2/29/2020 period, we used information from the earliest one.

### 2.3. World Trade Center Exposure

World Trade Center exposure information was obtained from participants’ first post-9/11 health monitoring questionnaire. We categorized rescue/recovery workers who first arrived at the WTC site on the morning of 9/11 as having had high-intensity exposure, those who arrived at the site on the afternoon of 9/11 or on 9/12/2001 as having moderate exposure, and those who arrived between 9/13-9/24/2001 as having low exposure [26].

### 2.4. Post-traumatic Stress Disorder Symptoms and Depressive Symptoms

Starting in 2005, the FDNY health monitoring questionnaire included validated screening instruments for PTSD and depression. The 17-item PTSD Checklist Specific (PCL-S) [27,28,29] and a modified version of the Life Events Checklist [30] were used to assess PTSD symptoms and participants’ most traumatic events, respectively, as detailed in our previous study [6]. Depressive symptoms were assessed via the Center for Epidemiologic Studies Depression Scale (CES-D) [31]. Higher scores indicated greater symptom severity [27,31]. We used the PCL-S and CES-D results from individuals’ first health monitoring questionnaire of the 3/1/2018-2/29/2020 time period in our analyses; these were included in regression models first as continuous scores and then as binary variables. PCL-S scores ≥44 and CES-D scores ≥16 corresponded to positive screens for PTSD and for depression, hereafter referred to as “PTSD” and “depression”, respectively.

### 2.5. Subjective Cognitive Function

Items probing self-perception of cognitive functioning were assessed within the health monitoring questionnaires, at the same time as PTSD symptoms and depressive symptoms. We used the Cognitive Function Instrument (CFI), a validated measure of self-perceived cognitive and functional change over the past year [23,24,25,32,33]. Longitudinal studies have shown CFI scores to be predictive of cognitive decline in older adults without clinical dementia at baseline [23,33,34]. CFI items can be classified as primarily addressing either cognitive abilities, such as memory, or functional abilities, such as using everyday appliances [23]. Participants responded “Yes”, “Maybe”, or “No” to each of the 14 items on the CFI and received scores of either 1, 0.5 or 0 points for each answer, as previously described [6]. When calculating CFI scores for the current analyses, we excluded participant responses to a question about social isolation, because a similar question exists on both the PCL-S and the CES-D. Scores, therefore, had a possible range of 0–13 points; higher scores corresponded to greater subjective cognitive change.

### 2.6. Statistical Analyses

Demographics, work assignment on 9/11, WTC exposure, smoking status, and PTSD and depression status at the time of CFI completion were assessed and presented as proportions (*n*, %) and means (±SD). We performed a multivariable linear regression analysis to estimate the association between WTC exposure level, included as an ordinal variable, and CFI score, controlling for the potential confounders of age at time of health monitoring exam, sex, race, education level, work assignment on 9/11, and smoking status. To determine whether the association of WTC exposure level with CFI score was mediated by either PCL-S score or CES-D score, we used a method developed by Vanderweele et al. [35,36,37,38], who expanded on the approach originally outlined by Baron and Kenny [39]. This method involved the following regression models for the mediator and outcome, respectively [35]:

(1) $$\begin{array}{l} E\left[M_{i}|a_{i},c_{i}\right]=\beta_{0}+\beta_{1}a_{i}+{\beta^{\prime }}_{2}c_{i}\\ E\left[Y_{i}|a_{i},m_{i},c_{i}\right]=\theta_{0}+\theta_{1}a_{i}+\theta_{2}m_{i}+\theta_{3}a_{i}m_{i}+{\theta^{\prime }}_{4}c_{i} \end{array}$$

**Figure 1:**
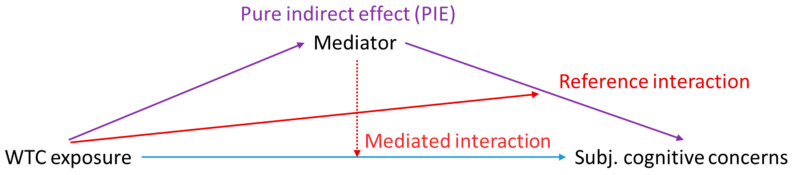
Four-way Decomposition of the Total Effect of World Trade Center Exposure on Subjective Cognitive Concerns. The solid blue line represents the controlled direct effect, or the effect of World Trade Center (WTC) exposure on Cognitive Function Instrument (CFI) score when the mediator is absent. The solid purple lines represent the pure indirect effect, or the component of the total effect of WTC exposure on CFI score that is a result of mediation (and not interaction). The solid red line represents the reference interaction, which is the component of the total effect resulting from interaction and not mediation, and the dotted red line represents the mediated interaction, which is the component resulting from both mediation and interaction.

Here, *Y_i_* represents the score on the CFI, *a_i_* represents the exposure level, *m_i_* the mediator, and *c_i_* a vector of covariates for study participant *i*. The components of mediation and interaction (Figure 1) are defined for a one-unit difference in the WTC exposure level with the following four-way decomposition [22]:

(2) $$\begin{array}{l} E[CDE|c_{i}]=\theta_{1}\\ E[INTref|c_{i}]=\theta_{3}\left(\beta_{0}+{\beta^{\prime }}_{2}c_{i}\right)\\ E[INTmed|c_{i}]=\theta_{3}\beta_{1}\\ E[PIE|c_{i}]=\theta_{2}\beta_{1} \end{array}$$

The controlled direct effect (*CDE*) is defined as the effect of the exposure on the outcome when the mediator is absent; the reference interaction (*INT_ref_*) is the component that is a result of interaction and not mediation; the mediated interaction (*INT_med_*) is the component resulting from both interaction and mediation; and, finally, the pure indirect effect (*PIE*) is the component of the effect of the exposure on the outcome that can be attributed to mediation and not interaction [36,40]. The total effect (*TE*) is the sum of these four components. The percentage of the total effect that was mediated by PCL-S score or by CES-D score was estimated using the formula (*PIE* + *INT_med_*)/TE, and is of primary interest. As the vast majority of individuals with PTSD symptoms in our cohort had concurrent depressive symptoms [6], we assessed these two potential mediators in separate models. We used the delta method to estimate confidence intervals.

After performing these mediation analyses in the full study population of *N* = 9516, we examined whether mediation of the relationship between WTC exposure and CFI score by PTSD symptoms and/or depressive symptoms varied based on age. We stratified the population into two subgroups defined by participant age at the time of CFI completion. The regression models described above were rerun using data from only younger (aged 36–55 years) and then from older (aged > 55 years) participants. The same models were also re-run using data from individuals aged > 65 at the time of CFI completion only.

Sensitivity analyses involved the inclusion of binary PTSD (PCL-S score ≥ 44) and depression (CES-D score ≥ 16) variables in the models instead of continuous PCL-S and CES-D scores. Finally, we assessed whether depressive symptoms mediated the association between WTC exposure level and CFI score independent of PTSD symptoms by excluding individuals who scored ≥ 44 on the PCL-S.

The regression models included the covariates age, sex, race, education level, work assignment on 9/11, and smoking status. Some of these covariates, such as work assignment on 9/11, are associated with WTC exposure and PTSD/depressive symptoms [19,20,21]; others are associated with cognitive function [11,41,42]. All analyses were conducted using SAS version 9.4. Reported *p*-values are two-sided and considered statistically significant when <0.05.

## 3. Results

### 3.1. Population Characteristics

Table 1 contains demographic and other characteristics of the final study population, displayed as proportions (%) or mean ± SD. Participant age at the time of CFI completion ranged from 36 to 83 years (mean ± SD: 56.6 ± 7.6 years). The CFI scores ranged from 0 to 13 points (median [IQR]: 0 [0–2]), after excluding participant responses to the question about social isolation. Mean PCL-S and CES-D scores at the time of CFI completion were 25.1 ± 11.0 (range: 17–85) and 8.7 ± 9.2 (range: 0–60), respectively.

Of 8883 participants who reported experiencing at least one traumatic event in their lifetimes via the Life Events Checklist, 78% (6962/8883) listed WTC rescue/recovery work as their most traumatic experience. Eighty-three percent (661/796) of the subpopulation who screened positive for PTSD reported that WTC rescue/recovery work was their most traumatic experience.

### 3.2. WTC Exposure and CFI Score

Table 2 displays the results of a multivariable linear regression that modeled the relationship between WTC exposure level and CFI score. Greater WTC exposure was associated with increasing CFI score (β: 0.25; 95% CI: 0.17, 0.34; *p* < 0.001), controlling for age, sex, race, education level, work assignment on 9/11, and smoking status. Individuals with high-level WTC exposure would, therefore, have CFI scores 0.5 points higher, on average, than those with low-level exposure.

### 3.3. Mediators of the Association between WTC Exposure and CFI Score

Table 3 shows the results of mediation analyses examining whether PTSD symptoms or depressive symptoms mediated the WTC exposure–CFI score relationship in the full study population. The association of WTC exposure level with CFI score was entirely mediated by PCL-S score (percent mediated: 111.0%; 95% CI: 83.1, 138.9), when controlling for age, sex, race, education level, work assignment, and smoking status. When CES-D score was included in the regression models in place of PCL-S score, the results also showed CES-D score to be a substantial and statistically significant mediator of the WTC exposure–CFI score association (percent mediated: 82.1%; 95% CI: 60.6, 103.7), after controlling for the covariates listed above.

### 3.4. Secondary Analysis

In Table 4 and Table 5, results from our age group-stratified mediation analyses are shown; these were similar to the results of the primary analyses presented above. Notably, PCL-S score entirely mediated the association between WTC exposure level and CFI score in both younger (aged ≤ 55; *N* = 4356) (Table 4) and older participants (aged > 55; *N* = 5160) (Table 5). While CES-D score was a significant mediator in both age groups, the mediation was greater in younger vs. older individuals (percent mediated: 92.2%; 95% CI: 55.5, 128.8 vs. 73.1%; 95% CI: 47.2, 99.0, respectively).

When we restricted the mediation analyses to the subpopulation of older adults >65 years old (*N* = 1147), we again found that PCL-S score fully mediated the WTC exposure–CFI score association (percent mediated: 105.9%; 95% CI: 34.4, 177.5), and that CES-D score partially mediated the association (percent mediated: 63.2%; 95% CI: 12.6, 113.8).

### 3.5. Sensitivity Analyses

After substituting the binary PTSD and depression variables for continuous PCL-S and CES-D scores, we observed that PTSD symptoms (PCL-S score ≥ 44) and depressive symptoms (CES-D score ≥ 16) remained significant mediators of the association between WTC exposure level and CFI score, though the levels of mediation were lower compared with those observed in our primary analyses (percent mediated: 61.9%; 95% CI: 42.8, 81.1 and 60.6%; 95% CI: 41.9, 79.3 for PTSD and depression, respectively).

Finally, CES-D score remained a statistically significant mediator of the WTC exposure–CFI score relationship even in a sensitivity analysis that excluded individuals with PTSD (N included = 8720) (percent mediated: 65.5%; 95% CI: 23.1, 108.0).

## 4. Discussion

In our previous research, we observed an association between high-intensity WTC exposure and subjective cognitive concerns in FDNY rescue/recovery workers, as well as strong associations between both PTSD symptoms and depressive symptoms and subjective cognitive concerns [6]. Given the established relationship between WTC exposure and PTSD and depressive symptoms in this cohort [19,20,21,22], we sought to determine whether these mental health conditions were mediators of the more recently identified association of WTC exposure with subjective cognitive concerns. The results of our mediation analyses indicate that PTSD symptoms completely mediated the association between WTC exposure level and self-reported cognitive decline assessed via CFI score. Depressive symptoms, when assessed as a mediator, significantly mediated the WTC exposure–CFI score association, particularly in younger individuals. In sensitivity analyses, in which probable PTSD and depression were treated as binary variables, the levels of mediation remained high. Goodness-of-fit checks confirmed that even levels of PTSD symptoms or depressive symptoms below the threshold for PTSD or depression were associated with increased score on the CFI.

Our results demonstrate that increased subjective cognitive concerns in the most highly WTC-exposed group vs. the lesser exposed group can be attributed to elevated levels of PTSD and depression, which are well-established risk factors for cognitive dysfunction [8,9,10,11,12,13,41,43,44]. In other words, mental health outcomes associated with greater WTC exposure, rather than WTC exposure alone, increase workers’ risk for subjective cognitive concerns. While many studies linking PTSD and/or depression to cognitive decline or dementia have been performed in older populations [11,12,41,42,43], there is some evidence of an association between these mental health conditions and cognition in younger age groups as well [8,9,10,13,45]; for example, PTSD and depression were associated with worse cognitive outcomes in middle-aged military veterans [8,9] and general populations [13,45]. Additionally, investigations that assessed self-perception of cognitive functioning in young and middle-aged veterans have similarly found associations between PTSD symptoms and/or depressive symptoms and self-perceived cognitive impairment, despite using different subjective cognitive measures than the current study—i.e., the Neurobehavioral Symptom Inventory and the Everyday Memory Scale [17,18]. Because PTSD symptoms and depressive symptoms were assessed at the same time point as subjective cognitive concerns in the current study, further investigation of the pathways between these mental health conditions and cognition will be needed in this population.

The effects of WTC exposure on subjective cognitive and functional concerns as measured by the CFI were modest, equivalent to one additional item being endorsed as “Yes” rather than “Maybe” or “Maybe” rather than “No” in the highest intensity WTC exposure group vs. the lowest exposure group. Our prior study found that FDNY rescue/recovery workers more frequently endorsed CFI items related to cognition than items related to functional abilities [6]. However, the CFI may be a less sensitive measure of cognition than objective neuropsychological testing, and future research will examine the association of WTC exposure with objective cognitive measures in this cohort. An earlier study in a different population of WTC-exposed first responders did estimate this association, using the online neuropsychological battery Cogstate to assess cognitive function [7], and found that WTC exposure and PTSD symptom severity were both associated with worse cognitive outcomes. These previous investigations in WTC-exposed workers did not involve mediation analyses, and instead assessed WTC exposure and PTSD symptoms as independent predictors of objective or subjective cognition.

The very high levels of mediation by PTSD symptoms and depressive symptoms that we observed in our current study do not rule out the possibility that other potential causal pathways exist, and in particular do not rule out a biological substrate for the modest effects seen. Effects were similar in younger (≤55 years old) and older members of the cohort, which might argue against this being a precursor to typical late life neurodegenerative conditions such as Alzheimer’s disease or vascular dementia. Future research using biomarkers of neurodegeneration and vascular brain pathology could confirm this possibility. Other potential biological factors that could be on the causal pathway between WTC exposure intensity and cognitive change include sleep disorders, pulmonary function impairment [46], and inflammatory serum biomarkers associated with cognitive impairment [47]. Longitudinal analyses would be needed to confirm cross-sectional results to support causality. Without detracting from the need for such investigations, however, we emphasize that PTSD and depression are potentially modifiable disorders. Our observation that PTSD symptoms and depressive symptoms, alone or in association, entirely mediate the association of WTC exposure intensity with subjective cognitive change, raising the possibility that early recognition and effective treatment of PTSD and depression may improve cognitive outcomes.

This research has a number of strengths. Most members of the FDNY WTC-exposed cohort (9516/11,483; 83%) presented for routine medical monitoring between 3/2018 and 3/2020 and completed the CFI, making it unlikely that selection bias is affecting our results. Secondly, information on potential confounders was available via the monitoring questionnaire data and the FDNY employee database. WTC exposure was assessed years prior to the assessment of PTSD, depression, or subjective cognition. Finally, the entire range of outcomes on all three instruments was used in the analyses, thus including potential effects of what would be considered subclinical levels of PTSD symptoms, depressive symptoms, or cognitive impairment.

A limitation of this study was that we lacked information on participants’ pre- and early post-9/11 (baseline) cognitive function, PCL-S scores, and CES-D scores. Our inclusion of only firefighters and EMS who were active FDNY employees on 9/11 meant that the study population was largely free of objective cognitive impairment at baseline. While the possibility of residual confounding exists, as in all observational studies, workers’ WTC exposure level was determined by their assigned location on 9/11 rather than the participants’ voluntary choice; therefore, significant differences in the three groups’ pre-9/11 mental health statuses are unlikely.

Additional limitations include our exclusive use of subjective rather than objective cognitive and function measures, and a high level of PTSD and depression comorbidity that precluded both from being examined as concurrent mediators. Although we performed a mediation analysis assessing depressive symptoms independently from PTSD symptoms by excluding participants with probable PTSD, and still found depressive symptoms to be a significant mediator, we could not repeat this sensitivity analysis assessing PTSD symptoms as the mediator because 92% (734/796) of participants who screened positive for PTSD also screened positive for depression.

## 5. Conclusions

We observed that, while WTC exposure was modestly associated with subjective cognitive concerns, PTSD symptoms and depressive symptoms entirely mediated this association. In the absence of PTSD or depressive symptoms, WTC exposure would not be expected to be associated with subjective cognitive concerns in FDNY rescue/recovery workers after adjusting for age and other confounders. This study provides support for the need to recognize and treat PTSD and depression in individuals with self-perceived cognitive decline, even in those with levels of PTSD or depressive symptoms that do not reach the threshold for clinical diagnoses. Clinical trials are needed to determine whether such treatment can mitigate the impact of these mental health conditions on cognitive function.

## Figures and Tables

**Table 1:** Population characteristics.

Variable	Study Population *N* = 9516
Age on 9/11	39.5 ± 7.6
Age at subjective cognitive assessment, *N* (%)	
30–39	71 (0.8)
40–49	1709 (18.0)
50–59	4274 (44.9)
60–69	3004 (31.6)
70–79	446 (4.7)
80+	12 (0.1)
Sex, *N* (%)	
Male	9286 (97.6)
Female	230 (2.4)
Race, *N* (%)	
White	8518 (89.5)
Black	400 (4.2)
Hispanic	541 (5.7)
Other	57 (0.6)
Education level, *N* (%)	
High school	1766 (18.6)
Some college	3942 (41.4)
Associate’s Degree	1125 (11.8)
Bachelor’s Degree	2683 (28.2)
World Trade Center site arrival time, *N* (%)	
Morning of 9/11	1608 (16.9)
Afternoon on 9/11–9/12	6562 (69.0)
9/13–9/24	1346 (14.1)
Work assignment on 9/11, *N* (%)	
Firefighter	8333 (87.6)
EMS	1183 (12.4)
Smoking status, *N* (%)	
Never	6265 (65.8)
Former	2948 (31.0)
Current	303 (3.2)
Potential mediators, Mean ± Std. Dev.	
Current PCL score	25.1 ± 11.0
Current CES-D score	8.7 ± 9.2
Potential mediators, *N* (%)	
PTSD (PCL ≥ 44)	796 (8.4)
Depression (CES-D ≥ 16)	1630 (17.1)

**Table 2:** Linear regression analysis of World Trade Center (WTC) exposure intensity predicting Cognitive Function Instrument (CFI) score in FDNY rescue/recovery workers.

Variable	β	95% Confidence Interval	*p*
Higher-level WTC exposure	0.25	0.17, 0.34	<0.001
Age	0.02	0.01, 0.02	<0.001
Higher education level	−0.14	−0.18, −0.09	<0.001
Work assignment on 9/11			
EMS	0.09	−0.08, 0.26	0.2845
Firefighter	Ref	Ref	Ref
Smoking status			
Current smoker	0.03	−0.24, 0.30	0.8419
Former smoker	0.18	0.07, 0.28	0.001
Never smoker	Ref	Ref	Ref
Race			
Black	0.09	−0.15, 0.34	0.4480
Hispanic	0.35	0.13, 0.56	0.0015
Other	0.65	0.05, 1.26	0.0346
White	Ref	Ref	Ref
Sex			
Female	0.43	0.10, 0.76	0.0106
Male	Ref	Ref	Ref

**Table 3:** Mediators of the association between WTC exposure level and CFI score in FDNY rescue/recovery workers.

Mediator	Total Effect (95% CI)	Percentage Mediated (95% CI)	*p*	Four-Way Decomposition		*p*
**PCL-S Score**	0.2572 (0.1736, 0.3408)	111.0 (83.1, 138.9)	<0.001	CDE (95% CI)	−0.0271 (−0.0912, 0.0370)	0.4069
				INT_ref_ (95% CI)	−0.0011 (−0.0068, 0.0046)	0.7090
				INT_med_ (95% CI)	0.0022 (−0.0092, 0.0135)	0.7087
				PIE (95% CI)	0.2833 (0.2286, 0.3379)	<0.001
**CES-D Score**	0.2536 (0.1702, 0.3370)	82.1 (60.6, 103.7)	<0.001	CDE (95% CI)	0.0534 (−0.0113, 0.1180)	0.1058
				INT_ref_ (95% CI)	−0.0081 (−0.0133, −0.0028)	0.0026
				INT_med_ (95% CI)	0.0161 (0.0067, 0.0255)	0.001
				PIE (95% CI)	0.1921 (0.1410, 0.2433)	<0.001

**Table 4:** Mediators of the association between WTC exposure level and CFI score in FDNY rescue/recovery workers aged ≤ 55.

Mediator	Total Effect (95% CI)	Percentage Mediated (95% CI)	*p*	Four-Way Decomposition		*p*
**PCL-S Score**	0.2436 (0.1221, 0.3650)	124.4 (75.7, 173.0)	<0.001	CDE (95% CI)	−0.0527 (−0.1465, 0.0410)	0.2703
				INT_ref_ (95% CI)	−0.0067 (−0.0159, 0.0025)	0.1553
				INT_med_ (95% CI)	0.0133 (−0.0046, 0.0313)	0.1461
				PIE (95% CI)	0.2896 (0.2121, 0.3671)	<0.001
**CES-D Score**	0.2397 (0.1185, 0.3609)	92.2 (55.5, 128.8)	<0.001	CDE (95% CI)	0.0296 (−0.0644, 0.1236)	0.5373
				INT_ref_ (95% CI)	−0.0108 (−0.0194, −0.0021)	0.0149
				INT_med_ (95% CI)	0.0215 (0.0065, 0.0365)	0.0049
				PIE (95% CI)	0.1993 (0.1258, 0.2729)	<0.001

**Table 5:** Mediators of the association between WTC exposure level and CFI score in FDNY rescue/recovery workers aged > 55.

Mediator	Total Effect (95% CI)	Percentage Mediated (95% CI)	*p*	Four-Way Decomposition		*p*
**PCL-S Score**	0.2696 (0.1545, 0.3848)	100.1 (67.3, 132.8)	<0.001	CDE (95% CI)	−0.0035 (−0.0913, 0.0844)	0.9384
				INT_ref_ (95% CI)	0.0033 (−0.0042, 0.0108)	0.3863
				INT_med_ (95% CI)	−0.0066 (−0.0215, 0.0082)	0.3820
				PIE (95% CI)	0.2764 (0.1997, 0.3532)	<0.001
**CES-D Score**	0.2666 (0.1517, 0.3814)	73.1 (47.2, 99.0)	<0.001	CDE (95% CI)	0.0776 (−0.0115, 0.1666)	0.0878
				INT_ref_ (95% CI)	−0.0058 (−0.0121, 0.0006)	0.0758
				INT_med_ (95% CI)	0.0115 (−0.0002, 0.0233)	0.0542
				PIE (95% CI)	0.1832 (0.1122, 0.2543)	<0.001
